# Next Generation Sequencing Analysis of Biofilms from Three Dogs with Postoperative Surgical Site Infection

**DOI:** 10.1155/2014/282971

**Published:** 2014-12-10

**Authors:** L. M. König, R. Klopfleisch, D. Höper, A. D. Gruber

**Affiliations:** ^1^Department of Veterinary Pathology, College of Veterinary Medicine, Freie Universitaet Berlin, 14163 Berlin, Germany; ^2^Friedrich-Loeffler-Institute, Federal Research Institute for Animal Health, Greifswald, 17498 Insel Riems, Germany

## Abstract

The composition of biofilms in chronic wound infections of dogs is unclear. In the present study, histologically identified biofilms attached to sutures in chronically infected wounds of three dogs were examined by next generation sequencing of total DNA extracted from formalin-fixed and paraffin-embedded tissue samples. The analysis identified an inhomogeneous bacterial composition in three tissues containing biofilms. Some of the identified bacterial families such as *Staphylococci* and *Streptococci* have been found before in biofilms associated with human and canine wounds but in this study were quantitatively in the minority. The majority of the reads classified as bacterial sequences had the highest identity with sequences belonging to the Porphyromonadaceae, Deinococcaceae, Methylococcaceae, Nocardiaceae, Alteromonadaceae, and Propionibacteriaceae and thus taxons of so far minor relevance in veterinary medicine.

## 1. Introduction

Biofilms are accumulations of microorganisms which are attached to surfaces and embedded in a polymeric matrix in contrast to free-floating planktonic bacteria [1]. Biofilms receive increasing attention in human medicine due to postoperative surgical site infections after implantation of medical devices which in up to 54% of cases may contain biofilms [2]. In contrast to these results, we recently identified a much lower prevalence of biofilms on surgical suture segments in wounds of dogs, cats, and horses [3]. Their significance and factors that modulate their genesis and relevance are largely unknown.

The bacterial families and genera involved in the formation of biofilms on implanted medical devices significantly differ among the different studies in human and the only veterinary study [4]. Bacteria most commonly identified by culturing of biofilms on human material are* Staphylococcus epidermidis*,* Staphylococcus aureus*, and* Pseudomonas aeruginosa* [5]. However, a greater proportion of bacteria organized in biofilms are not cultivable by common microbiological culture techniques [6]. 16S rRNA sequencing has thus been suggested as a promising approach for the characterization of biofilm composition [7].

The present study aimed at identifying the composition of biofilms on suture material in chronically infected wounds of dogs using next generation sequencing (NGS) on formalin-fixed, paraffin-embedded (FFPE) tissue samples. Biofilms were identified in FFPE tissue samples submitted for routine diagnostic examination as recently described [8]. Briefly, biofilms were defined by three criteria, presence of suture material, an attached periodic-acid-Schiffs-reaction (PAS) positive polymeric matrix, and gram- and Giemsa-stainable bacteria.

## 2. Materials and Methods

Bacterial DNA was purified from FFPE tissue sections using a commercial kit (NucleoSpin Kit, Macherey-Nagel, Düren, Germany) as recently described [9, 10]. A total of 8100 ng of total DNA was extracted and used for NGS. Purified DNA was fragmented by sonication (M220 Focused-Ultrasonicator; Covaris, Woburn, Massachusetts, USA) and 500 ng of the fragmented DNA was used as input for library preparation with the aid of a SPRI-TE instrument (Beckman Coulter, Krefeld, Germany) with SPRIworks II cartridges and NEXTflex-96 DNA Barcodes (Bioo Scientific, Austin, TX, USA). Library preparation was done without automatic size selection and the resultant libraries were instead manually size selected (peak size 500 bp) with Ampure XP Beads (Beckman Coulter). Finally, the size selected libraries were quantified using the KAPA Library Quantification Kit, Illumina/Universal (KAPA Biosystems, Cape Town, South Africa) and sequenced with an Illumina MiSeq instrument (MiSeq Reagent Kit v2 (500 cycle); Illumina, San Diego, USA). The raw reads were analyzed using RIEMS (zit).

To clarify relevant results of DNA sequences and associated bacterial families, a deliberate mark of all families with a quantity of >40 reads was set and these families were selected (Table 1).

## 3. Results

**Figure 1:**
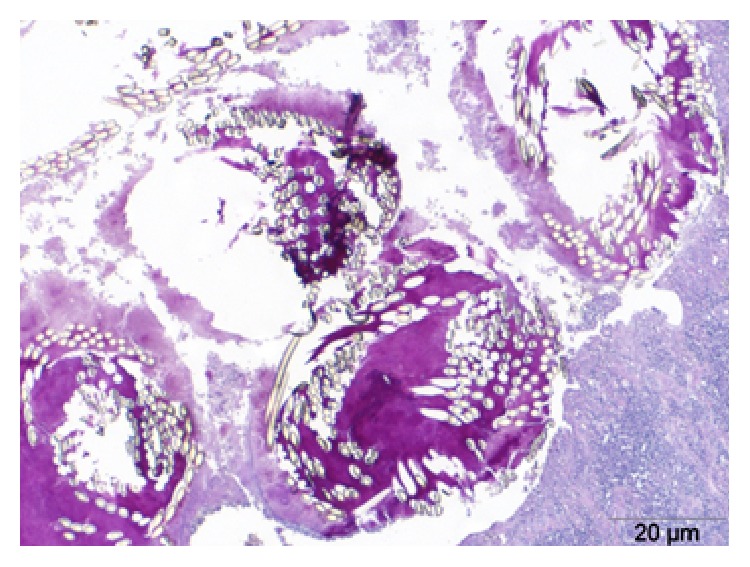
Case 1: PAS-positive extracellular polymeric matrix of a biofilm associated with polyfilic suture material, PAS-reaction.

The tissue sample with biofilm one was derived from an ovariohysterectomized uterus stump which included polyfilic suture segments of an adult female Jack-Russell (Figure 1). Histopathology revealed a chronic-active, lymphoplasmacytic, and granulomatous inflammation, and a biofilm associated with the suture material. NGS resulted in 1625631 high quality reads of which approximately two-thirds were classified as host sequences and roughly 12.5% could not be classified by similarity at the nucleic acid sequence level. Of the remainder, 12905 reads (0.8%) were assigned as bacterial sequences. The assigned families are shown in Table 1. The most prominent families were Porphyromonadaceae, Fusobacteriaceae, and Peptostreptococcaceae, representing 73% of all identified bacterial sequences.

The tissue sample with biofilm two was derived from a postcastration skin wound with polyfilic suture segments associated in an adult female Yorkshire Terrier. NGS obtained 360974 high quality reads of which approximately one-third were classified as host sequences. 11,610 reads (3.2%) were assigned as bacterial reads. The most prominent families were Deinococcaceae, Methylobacteriaceae, and Nocardiaceae representing 16.2% of all identified bacterial sequences.

The tissue with biofilm three was derived from a surgical skin wound with polyfilic suture segments associated in an adult, female Labrador Retriever. NGS obtained 1,459,690 high quality reads of which approximately 50% were classified as host sequences and 649 reads (0.1%) were assigned as bacterial reads. The most prominent families were Porphyromonadaceae, Alteromonadaceae, and Fusobacteriaceae, representing 37.8% of all identified bacterial sequences.

**Figure 2:**
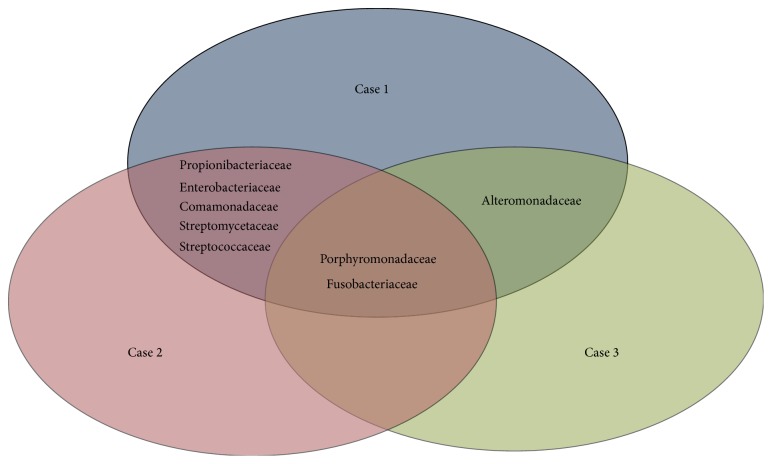
Bacterial families with overlapping presence in the three tissues with biofilms.

In a comparison regarding bacterial families, it is conspicuous that Fusobacteriaceae and Porphyromonadaceae were present in all three cases (Figure 2). In addition, Porphyromonadaceae and Alteromonadaceae were present in cases one and three. There were concordant DNA sequences of several bacterial families present in cases one and two, that is, Propionibacteriaceae, Streptomycetaceae, Streptococcaceae, Enterobacteriaceae, Comamonadaceae, and Flavobacteriaceae. In contrast, Deinococcaceae, Methylococcaceae, and Nocardiaceae were the most prominent bacterial families in case two. Nevertheless, Propionibacteriaceae, Methylobacteriaceae, Lactobacillaceae, and Streptococcaceae which were present in tissues of dogs number 1 and number 2 have also been detected in the previous report on biofilms from wounds of a single dog (Figure 2) [4].

The composition of bacterial species present in the three tissues with biofilms only partly overlapped, except for case number 3 which only contained species that were also present in case 1 (Figure 2). In contrast, cases number 1 and number 2 only partly overlapped in bacterial species and cases number 2 and number 3 did not overlap at all.* Tannerella forsythia* was the most common bacterial species in cases number 1 and number 3; in number 2 it was* Deinococcus geothermalis* (Figure 2).

## 4. Discussion

Biofilms can be a single species microbial population or a community of multiple microbial species and may cover a vast array of abiotic and biotic surfaces [11]. The diagnosis of the composition of biofilms by the conventional culturing methods is however difficult. Recent studies compared culture techniques and molecular techniques to identify the bacterial composition of biofilms in wounds. They found that only 10% of the bacterial species in wounds can be identified using standard culture technique when compared to the higher number of species identified by molecular techniques [12]. It is thus not surprising that at least two of the biofilms analyzed in this study contained a wide variety of DNA sequences which could be attributed to numerous different bacterial species.

Porphyromonadaceae (*Tannerella forsythia*) was the most common bacterial family in biofilms one and three. In contrast, Deinococcaceae, Methylococcaceae, and Nocardiaceae were the most prominent bacterial families in the tissue with biofilm two. The composition of bacterial families present in the three tissues with biofilms was thus only partly overlapping, except for case three which only contained species that were also present in case one. Bacterial families which were present in two tissues with biofilms were Alteromonadaceae, Propionibacteriaceae, Streptomycetaceae, Streptococcaceae, Enterobacteriaceae, Comamonadaceae, and Flavobacteriaceae and may thus be interesting candidates for further analysis as initiators of chronic wound infection in dogs.

Comparison with the only recent study on the composition of wound-associated biofilms in dogs revealed partly overlapping bacterial species with the present study. Pyrosequencing of those biofilms detected Propionibacteriaceae, Methylobacteriaceae, Lactobacillaceae, and Streptococcaceae which were also present in biofilms one and two in our study [4].

So far only few studies on wound-associated biofilm composition using NGGS are available in human medicine. These studies however revealed that up to 60% of chronic wounds contained biofilms whereas acute wounds showed biofilm structures in only 6% of the cases. Molecular analysis also discovered a large variety of bacteria, including strictly anaerobic bacteria, which were not detectable by culture. Amongst others, molecular analysis identified Prevotellaceae, Pseudomonadaceae, Staphylococcaceae, and Porphyromonadaceae in these biofilms, all of which were also detected in the biofilms of the present study [13].

Extraction and sequencing of DNA from FFPE material by NGGS was successfully applied to identify bacterial species in chronic wounds. Obviously, NGGS is still by far too expensive for routinely examinations. The present study however shows that it nevertheless can give valuable additional information on the composition of the biofilm. Further studies however have to proof whether the bacterial species and families identified by the present approach are really present and viable in the wounds and thus contribute to the clinical symptom. The present study thus gives first hints towards which culturing media and conditions are necessary to analyze biofilms in chronic wounds of dogs.

Taken together our study shows that biofilms on suture material in chronic wounds of dogs may be composed of a wide variety of bacterial species which may not be covered by conventional bacterial culturing. Further studies however have to confirm which of the identified bacterial species are actually contributing to the morphologically identified biofilms and which are just bystander infections. Molecular methods like NGGS may thus be an alternative approach to identify the bacterial species involved in these chronic infections and to choose the correct therapeutic measures in the future.

## Figures and Tables

**(a):** 

	Biofilm # 1
5014	Porphyromonadaceae
4010	Fusobacteriaceae
399	Peptostreptococcaceae
325	Clostridiaceae
317	Bacteroidaceae
241	Streptococcaceae
146	Propionibacteriaceae
140	Flavobacteriaceae
95	Prevotellaceae
88	Rikenellaceae
72	Enterobacteriaceae
72	Alteromonadaceae
65	Desulfomicrobiaceae
58	Comamonadaceae
56	Spirochaetaceae
50	Bacillaceae
49	Leptotrichiaceae
46	Lachnospiraceae
43	Streptomycetaceae

**(b):** 

	Biofilm # 2
3219	Deinococcaceae
1954	Methylobacteriaceae
833	Nocardiaceae
598	Mycobacteriaceae
426	Corynebacteriaceae
375	Propionibacteriaceae
284	Pseudonocardiaceae
251	Gordoniaceae
228	Streptomycetaceae
184	Micrococcaceae
166	Streptococcaceae
151	Rhodocyclaceae
148	Sphingomonadaceae
142	Staphylococcaceae
136	Dietziaceae
127	Geodermatophilaceae
98	Micromonosporaceae
93	Xanthomonadaceae
84	Pseudomonadaceae
83	Cellulomonadaceae
79	Rhizobiaceae
75	Bradyrhizobiaceae
70	Microbacteriaceae
70	Nakamurellaceae
69	Burkholderiaceae
67	Fusobacteriaceae
63	Nocardioidaceae
62	Tsukamurellaceae
60	Porphyromonadaceae
59	Frankiaceae
51	Enterobacteriaceae
50	Dermacoccaceae
48	Lactobacillaceae
47	Nocardiopsaceae
47	Promicromonosporaceae
46	Comamonadaceae
42	Flavobacteriaceae
41	Moraxellaceae
40	Rhodobacteraceae

**(c):** 

	Biofilm # 3
163	Porphyromonadaceae
42	Alteromonadaceae
40	Fusobacteriaceae

## References

[1] Costerton J. W., Stewart P. S., Greenberg E. P. (1999). Bacterial biofilms: a common cause of persistent infections. *Science*.

[2] Edmiston C. E., Krepel C. J., Marks R. M. (2013). Microbiology of explanted suture segments from infected and noninfected surgical patients. *Journal of Clinical Microbiology*.

[3] König L., Klopfleisch R., Kershaw O., Gruber A. D. (2014). Prevalence of biofilms on surgical suture segments in skin wounds of dogs, cats and horses. *Veterinary Pathology*.

[4] Swanson E. A., Freeman L. J., Seleem M. N., Snyder P. W. (2014). Biofilm-infected wounds in a dog. *Journal of the American Veterinary Medical Association*.

[5] Freeman K., Woods E., Welsby S., Percival S. L., Cochrane C. A. (2009). Biofilm evidence and the microbial diversity of horse wounds. *Canadian Journal of Microbiology*.

[6] Safaee S., Weiser G. C., Cassirer E. F., Ramey R. R., Kelley S. T. (2006). Microbial diversity in bighorn sheep revealed by culture-independent methods. *Journal of Wildlife Diseases*.

[7] Nyvad B., Crielaard W., Mira A., Takahashi N., Beighton D. (2013). Dental caries from a molecular microbiological perspective. *Caries Research*.

[8] König L. (2013). Biofilms in dogs. *Veterinary Pathology*.

[9] Klopfleisch R., Weiss A. T. A., Gruber A. D. (2011). Excavation of a buried treasure—DNA, mRNA, miRNA and protein analysis in formalin fixed, paraffin embedded tissues. *Histology and Histopathology*.

[10] Weiss A. T. A., Delcour N. M., Meyer A., Klopfleisch R. (2011). Efficient and cost-effective extraction of genomic dna from formalin-fixed and paraffin-embedded tissues. *Veterinary Pathology*.

[11] Davey M. E., O’Toole G. A. (2000). Microbial biofilms: from ecology to molecular genetics. *Microbiology and Molecular Biology Reviews*.

[12] Dowd S. E., Sun Y., Secor P. R. (2008). Survey of bacterial diversity in chronic wounds using Pyrosequencing, DGGE, and full ribosome shotgun sequencing. *BMC Microbiology*.

[13] James G. A., Swogger E., Wolcott R. (2008). Biofilms in chronic wounds. *Wound Repair and Regeneration*.

